# Patients who do not complete cardiac rehabilitation have an increased risk of cardiovascular events during long-term follow-up

**DOI:** 10.1007/s12471-020-01413-1

**Published:** 2020-03-20

**Authors:** M. Sunamura, N. ter Hoeve, R. J. G. van den Berg-Emons, E. Boersma, M. L. Geleijnse, R. T. van Domburg

**Affiliations:** 1 Capri Cardiac Rehabilitation, Rotterdam, The Netherlands; 2 Department of Rehabilitation Medicine, Erasmus University Medical Center, Rotterdam, The Netherlands; 3 Department of Cardiology, Thorax center, Erasmus University Medical Center Rotterdam, Rotterdam, The Netherlands

**Keywords:** Acute coronary syndrome, Lifestyle, Cardiac rehabilitation

## Abstract

**Background:**

Cardiac rehabilitation (CR) has favourable effects on cardiovascular mortality and morbidity. Therefore, it might reasonable to expect that incomplete CR participation will result in suboptimal patient outcomes.

**Methods:**

We studied the 914 post-acute coronary syndrome patients who participated in the OPTImal CArdiac REhabilitation (OPTICARE) trial. They all started a ‘standard’ CR programme, with physical exercises (group sessions) twice a week for 12 weeks. Incomplete CR was defined as participation in <75% of the scheduled exercise sessions. Patients were followed-up for 2.7 years, and the incidence of cardiac events was recorded. Major adverse cardiac events (MACE) included all-cause mortality, non-fatal myocardial infarction and coronary revascularisation.

**Results:**

A total of 142 (16%) patients had incomplete CR. They had a higher incidence of MACE than their counterparts who completed CR (11.3% versus 3.8%, adjusted hazard ratio [aHR] 2.86 and 95% confidence interval [CI] 1.47–5.26). Furthermore, the incidence of any cardiac event, including MACE and coronary revascularisation, was higher (20.4% versus 11.0%, aHR 1.54; 95% CI 0.98–2.44). Patients with incomplete CR were more often persistent smokers than those who completed CR (31.7% versus 11.5%), but clinical characteristics were similar otherwise.

**Conclusion:**

Post-ACS patients who did not complete a ‘standard’ 12-week CR programme had a higher incidence of adverse cardiac events during long-term follow-up than those who completed the programme. Since CR is proven beneficial, further research is needed to understand the reasons why patients terminate prematurely.

## What’s new?

- During a follow-up of 3 years, occurrence of major adverse cardiac events (MACE) was higher in patients with acute coronary syndrome who did not complete cardiac rehabilitation compared with those who did.
- Patients with incomplete cardiac rehabilitation were more often persistent smokers than those who did complete cardiac rehabilitation.

## Introduction

Cardiac Rehabilitation (CR) is a class I recommended intervention in patients with coronary artery disease and has beneficial effects on physical fitness, quality of life, cardiovascular risk factors and clinical outcome, including mortality [1–5]. Despite the evidence of these beneficial effects, CR programmes are still largely underutilised [6]. What’s more, a substantial number of patients that do participate in CR attend only a few sessions and then drop out prematurely. It might reasonably be expected that such suboptimal CR participation will in less favourable results as well. Suaya and Beauchamp were the first to describe a possible relation between the number of sessions attended and mortality [7, 8]. In more recent studies such a relation was also found in specific populations, including diabetic patients and women with coronary artery disease [9, 10]. However, contradictory findings of CR results were also reported [11], which may be caused by the lack of a unanimous definition of CR ‘participation’ and ‘completion’. For example, in several studies the attendance of at least one session was already regarded as CR participation [12, 13], whereas others used a more stringent definition [7, 8].

Recently, we presented the OPTImal CArdiac REhabilitation (OPTICARE) trial; a randomised controlled CR trial that enrolled 914 patients post acute coronary syndrome (ACS) [14, 15]. All patients started a ‘standard’ 12-week CR programme, and we aimed to assess an association between completion of the programme and incidence of adverse cardiac events during prolonged follow-up. Furthermore, we looked for possible determinants of CR completion. For the current analysis, CR is defined as ‘complete’ if the participant attended at least 75% of the physical programme, and ‘incomplete’ if otherwise.

## Material and methods

### Patients

OPTICARE was an open, randomised, controlled trial that studied the effects of intensified and prolonged CR compared with standard CR on cardiac risk profile, levels of daily physical activity, quality of life and health care consumption in patients after ACS. Details on inclusion and exclusion criteria and study procedures are described in the OPTICARE design paper [14]. Briefly, 914 patients who were discharged after ACS admission were scheduled to receive ‘standard’ CR (see below) for 12 weeks according to the European Society of Cardiology (ESC) guidelines [16]. A total of 608 patients were randomised to receive extended CR with extra behavioural counselling in individual telephone sessions or group sessions until 9 months after completion of standard CR. The primary outcome was the SCORE (Systematic COronary Risk Evaluation) 10-year cardiovascular mortality risk function at 18 months after the index ACS. Results of OPTICARE showed no additional benefits with respect to SCORE in patients randomised to extended CR [15]. Patients largely reached target levels of modifiable risk factors already following standard CR. Therefore, for the purpose of the current analysis, all OPTICARE patients were analysed as a homogeneous cohort. The study was performed according to the CONSORT guidelines. The study was conducted according to the Helsinki Declaration and the study was approved by the Medical Ethics Committee of the Erasmus MC. All patients consented to participation in this study.

### CR according to the Capri programme

CR was offered by Capri Cardiac Rehabilitation (Capri CR) at eight different locations in the cities of Rotterdam and The Hague with referrals from 10 hospitals in the broader Rotterdam/The Hague region. The core of the Capri CR programme is 1.5-hour group exercise sessions twice weekly. Besides the exercise programme, verbal and written instructions are given on how to deal with exercise, diet, smoking cessation and stress management. The aim of the programme is to improve adherence to lifestyle modification, and to help patients to adopt a positive role in the care of their own health. If necessary, individual consultations with a psychiatrist, psychologist, social worker and dietician are provided. A multidisciplinary team, led by a physician, specialised physiotherapists, nurses and social workers, together with the patient, determined the exact length of a CR programme with an average duration of 12 weeks. Upon completion of the CR programme, a maximum (symptom-limited) bicycle stress test is performed.

For the current analysis, CR is defined as ‘complete’ if the participant attended at least 75% of the physical programme, and ‘incomplete’ otherwise [8].

### Data collection

Data were collected on demographic variables, cardiovascular risk factors and cardiovascular history. Data on cardiovascular risk factors were measured, as these were part of the OPTICARE study endpoint (SCORE). Systemic arterial hypertension was defined as a systolic blood pressure >140 mm Hg and/or diastolic blood pressure >90 mm Hg, or treatment for hypertension. Hypercholesterolaemia was defined as a total cholesterol >6.0 mmol/l or treatment for hypercholesterolaemia before index event. Diabetes was diagnosed as a fasting plasma glucose >7.0 mmol/l or the use of glucose-lowering therapy medication. Smoking was defined as self-reported smoking at the index ACS. At randomisation, self-reported smoking cessation was verified by using a Smokerlyzer, which measures the concentration of carbon monoxide in breath. Family history of premature coronary artery disease was defined as a self-reported history of any first-degree family member with a history of myocardial infarction (MI), percutaneous coronary intervention (PCI), or coronary artery bypass grafting (CABG) before the age of 60 years.

### Clinical endpoints

For the current analysis, clinical endpoints were collected until DATE, which resulted in a median (IQR) follow-up of 2.7 (range 2 years to 5 years) after the index ACS. We defined the incidence of major adverse cardiovascular events (MACE) as our primary endpoint, which includes all-cause mortality, non-fatal MI and coronary revascularisation. We also collected data on other cardiovascular events, including hospitalisation for unstable angina (chest pain in rest with negative biomarkers but positive stress testing), stable angina (chest pain on exertion with negative biomarkers), non-specific chest pain (chest pain in rest with negative biomarkers and negative or absent stress testing), cardiac arrhythmia’s and heart failure. We also counted cardiac emergency room visits without hospitalisation. All clinical endpoints were verified by an independent Clinical Event Committee.

### Statistical analysis

Continuous variables were presented as mean (standard deviation), whereas categorical variables are expressed as numbers and percentages. Comparisons between patients with and without completed CR were performed by the Student’s t‑test for continuous variables, and Pearson’s chi-squared test or Fisher’s exact tests, for categorical variables.

The cumulative incidence of the clinical endpoints over time was studied using the Kaplan-Meier method, whilst log-rank tests were applied to evaluate differences between patients with and without completed CR. Patients lost to follow-up were considered at risk until the date of last contact, at which point they were censored.

Cox regression analyses were performed to further study whether or not completing the programme was associated with the incidence of study endpoints. We ran univariable models, and multivariable models with adjustment for outcome determinants and potential confounders. In view of the number of MACE (*N* = 45, see Results section) in the corresponding MACE model, we decided to adjust only for age, gender, diabetes, prior history of cardiovascular events, and randomly allocated study treatment, in order to avoid model overfitting [17]. With respect to any cardiovascular events, we adjusted for age, gender, prior cardiovascular event, diabetes, hypertension, familial history, smoking, hypercholesterolaemia and study treatment. Findings are presented as crude hazard ratios (HR) and adjusted hazard ratios (aHR) with 95% confidence intervals (CI).

Logistic regression analyses were performed to identify factors that were associated with CR incompletion. We considered the following factors: age, sex, prior cardiovascular event, diabetes, hypertension, family history of premature coronary artery disease, smoking and hypercholesterolaemia. Findings are presented as odds ratios (OR) with 95% CI.

All statistical tests were two-tailed and a *p*-value of <0.05 was considered statistically significant. Statistical analysis was performed with SPSS 24 for Windows (SPSS Inc., Chicago, Il, USA).

## Results

### Patient characteristics

In total, 770 (84%) patients completed CR, and 142 patients (16%) did not. Patients who completed CR had, on average, 23 exercise sessions, as compared with 6 sessions for those who did not complete CR (*p*-value <0.001). Patients with and without CR completion had similar baseline characteristics, except for smoking, and the use of statins and angiotensin-converting enzyme inhibitors: those who did not complete CR were more often persistent smokers (31.7% versus 11.5%, *p*-value <0.001) (Tab. 1).

**Table 1:** Patient characteristics in relation to completion and incompletion of CR

	Complete CR	Incomplete CR	Odds ratio ^a^	(95% CI)	*P*-value
No. of patients	770	142			
Age, years	57.6 (9.1)	55.5 (10.8)	0.98	(0.96–0.99)	0.11
Man	623 (80.9)	114 (80.3)	0.96	(0.61–1.50)	0.86
*Initial revascularisation treatment*					
PCI	600 (77.9)	112 (78.9)	1.06	0.68–1.63	0.80
CABG	3 (0.4)	2 (1.4)	3.65	0.60–22.05	0.13
No Revascularisation	167 (21.7)	28 (19.7)	1.10	0.71–1.72	0.74
*Cardiovascular risk factors*					
Family history	409 (53.1)	71 (50.0)	0.88	(0.62–1.26)	0.50
Diabetes	102 (13.2)	19 (13.4)	1.01	(0.60–1.71)	0.96
Hypertension	319 (41.4)	55 (38.7)	0.89	(0.62–1.29)	0.55
Smoking					
– Persistent	89 (11.5)	45 (31.7)	2.78	(1.70–4.55)	<0.001
– Never	461 (59.9)	57 (40.1)	0.68	(0.44–1.05)	0.082
– Quit (reference)	220 (28.5)	40 (28.1)	1		
Hypercholesterolaemia	268 (34.8)	44 (31.0)	0.84	(0.57–1.23)	0.38
*Cardiovascular history*	129 (16.8)	33 (23.2)	1.50	0.97–2.31	0.14
MI	63 (8.2)	17 (12.0)	1.53	0.86–2.69	0.14
PCI	72 (9.4)	17 (12.0)	1.32	0.75–2.31	0.33
CABG	9 (1.2)	4 (2.8)	2.45	0.74–8.07	0.13
CVA	3 (0.4)	2 (1.4)	3.65	0.60–21.05	0.13
TIA	15 (1.9)	4 (2.8)	145	0.48–4.46	0.50
*Cardiac medication*					
Anticoagulants	766 (99.4)	139 (97.9)	NA		0.68
Statins	740 (96.1)	128 (90.1)	0.42	0.20–0.88	0.017
ACE inhibitors	545 (70.8)	87 (61.3)	0.68	0.46–0.99	0.046
Beta blockers	634 (82.3)	113 (79.6)	0.91	0.57–1.45	0.69

Continuous data are presented as mean (standard deviation) values, and categorical data are presented as numbers (percentages)

*ACE* angiotensin-converting enzyme, *CABG* coronary artery bypass grafting, *CI* confidence interval, *CR* cardiac rehabilitation, *CVA* cerebrovascular accident, *MI* myocardial infarction, *PCI* percutaneous coronary intervention, *TIA* transient ischaemic attack

^a^ Odds ratio related with the characteristic for incompletion of the cardiac rehabilitation program

### CR incompletion and clinical endpoints

Forty-five patients (4.9%) had at least one MACE (Tab. 2). The incidence of MACE was higher in the patients who had incomplete CR than in their counterparts who completed the programme (11.3% versus 3.8%; HR 2.94 and 95% CI 1.59–5.55). After adjustment for multiple factors (see Methods section), this relation remained significant (aHR 2.86 and 95% CI 1.47–5.26).

**Table 2:** Cardiovascular events after CR

	Complete CR	Incomplete CR	*P*-Value
No. of patients	770	142	
Any MACE	29 (3.8)	16 (11.3)	
– Mortality	8 (1.0)	4 (2.8)	0.089
– ST-elevation MI	7 (0.9)	2 (1.4)	0.58
– Non-ST-elevation MI	10 (1.3)	4 (2.8)	0.17
– CABG	4 (0.5)	2 (1.4)	0.23
– PCI	9 (1.1)	9 (6.3)	0.003
Any non-major cardiovascular events	85 (11.0)	29 (20.4)	
– Hospitalisation because of			
– Unstable angina	4 (0.5)	2 (1.4)	0.23
– Stable angina	19 (2.5)	8 (5.6)	0.039
– Non-specific chest pain	17 (2.2)	6 (4.2)	0.16
– Arrhythmias	12 (1.5)	1 (0.7)	0.48
– Heart failure	0 (0.0)	0 (0.0)	–
– Cardiac emergency room visit without hospitalization	38 (4.9)	11 (7.7)	0.17

Data are presented as numbers (percentages)

*CABG* coronary artery bypass grafting, *CR* cardiac rehabilitation, *CVA* cerebrovascular accident, *MACE* major adverse cardiac event, *MI* myocardial infarction, *PCI* percutaneous coronary intervention

**Fig. 1:**
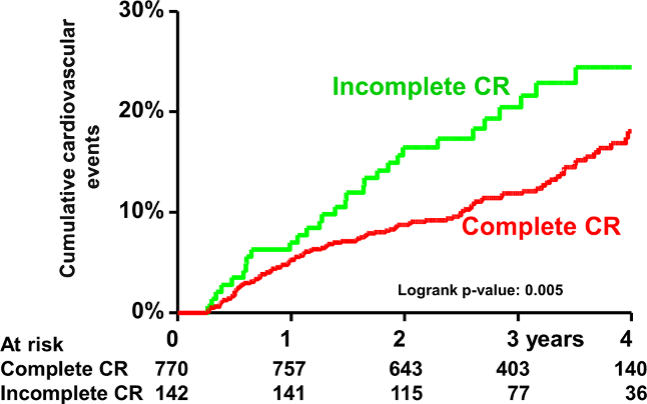
Cumulative incidence of any cardiovascular event. *CR* cardiac rehabilitation

In total, 114 patients (12.5%) suffered from one or more cardiovascular events (Tab. 2). The cumulative incidence of any cardiovascular event at 4 years in complete CR versus incomplete CR was 18% and 25% according to the Kaplan-Meier method, respectively (log-rank *p* = 0.006) (Fig. 1). Incomplete CR was associated with a higher incidence of cardiovascular events (HR 1.69; 95% CI 1.11–2.56). Again, after adjustment for multiple factors, this relation remained, although our criterion for statistical significance was not met (HR 1.54; 95% CI 0.98–2.44).

MACE during CR was not the reason for incomplete CR, as all 5 patients with MACE during CR ultimately completed CR after a new coronary intervention.

### Predictors of incomplete CR

Persistent smokers had 2.78 times higher odds of incomplete CR than those who quitted smoking since the ACS admission (OR 2.78 and 95% CI 1.70–4.55, *p*-value <0.001) (Tab. 1). Never-smokers tended to have lower odds of incomplete CR than quitters, but this difference was statistically non-significant. We could not identify any other characteristic that (independent of smoking behaviour) related with higher or lower odds of incomplete CR. The C‑index of the logistic regression model that related smoking behaviour to completion of the CR programme was 0.63. Thus, patients could not satisfactorily be identified as either completers or non-completers on the basis of their smoking behaviour alone.

## Discussion

We found that post-ACS patients who did not complete CR—defined as participation of at least 75% of the sessions—had an almost threefold higher incidence of MACE during prolonged follow-up. We further found that smoking behaviour was associated with the degree of CR completion—smoking cessation was a success factor. Still, the reasons why patients did not complete the 12-week ‘standard’ CR programme remained largely unknown.

Duration and intensity of CR programmes are highly variable [18]. In a systematic review of Rauch *et al. *[19] no comparison could be made between beneficial effects and the type or length of CR offered, given the wide heterogeneity of CR programmes which varied both in duration (3 weeks to 12 months) and intensity (2 to 5 sessions per week). Not surprisingly therefore, a definition of ‘complete CR’ is absent in the guidelines of the European Association for Cardiovascular Prevention & Rehabilitation [20] both in terms of length of a standard programme and in particular in terms of the number of attended sessions. Unfortunately, completion is not defined at all in most studies [12], whereas in other studies it ranged from minimal attendance of one session [13], to 50% in EUROASPIRE IV [21], and to 75% in the study of Beauchamp *et al. *[8]. We used the most stringent Beauchamp criterion, because in our opinion CR will only be effective if the absenteeism is minimal. This position is supported by our data, as incomplete CR according to Beauchamp was a strong and independent predictor of increased risk for cardiovascular events. It should be noticed that the majority of patients in the incomplete CR group quitted CR very early in the programme, resulting in an important difference in attended sessions: on average 6 versus 23 sessions. Patients who attended a number of sessions in-between were a minority. Therefore, in our opinion, the minimal number of sessions should be the chosen >75% of the physical programme, even though this is an arbitrary choice. In contrast to what one might expect, the occurrence of MACE during CR was not the reason for incomplete CR, as all 5 patients with MACE during CR ultimately completed CR after a new coronary intervention.

Benefits of complete versus incomplete CR were shown in a few retrospective studies. In 2009 Suaya* et al. *[7] was the first to show a relation between mortality and less than 25 attended CR sessions in elderly with coronary artery disease. Subsequently, these results were confirmed by a study of Beauchamp *et al. *in which patients who suffered from an acute myocardial infarction or who underwent PCI and attended less than 25% of the CR sessions had a more than two-fold increased mortality risk during 14-year follow-up, compared with those attended >75% of the sessions [8]. More recently, Armstrong *et al.* reported in a large study in almost 3000 diabetic patients, included between 1996 to 2010, that diabetic patients were less likely to start and to complete CR [9]. Although complete CR was not well defined, patients who fully participated in the 12-week CR programme had reduced mortality and hospitalisation in comparison with CR participants who did not complete CR. Finally, in a large study by Colbert *et al. *in over 6000 women with at least one-vessel coronary artery disease who participated in CR in 1996, complete CR, defined as at least 12 of 24 CR sessions (50%) including a 12-week post-CR assessment, had the lowest mortality during long-term follow-up [10].

In a recent retrospective study of our group we already demonstrated a reduction in 10-year mortality in ACS patients. We studied 1159 ACS patients who had primary PCI. We found that patients who attended a CR programme had a significantly lower 10-year mortality than their no-CR counterparts (14.7% versus 23.5%), and that patients who completed CR had a lower 10-year mortality compared with patients who started CR but did not complete the programme (13.6% versus 18.9%) [22]. The current study differs from the ones mentioned, because: 1) our patients constituted a more homogeneous population with ACS in the large majority treated with primary PCI; 2) medical therapy was more intense; and 3) medical therapy did not differ between the patients with complete and incomplete CR.

Since incompleteness of CR is associated with clinical outcome, it seems important that we identify patients at risk for drop-out. The single most important predictor for incompleteness of CR was smoking persistence. Other authors have also shown that smoking is an important predictor for incompletion of CR [23]. We can only speculate why. Patients are motivated but may simply not be able to quit for whatever reason. However, patients who are not motivated to quit smoking may also not be motivated to work on a healthier lifestyle in general either. It could also be that smoking is just part of other patient characteristics, such as a certain type of personality, limited education, and lower socio-economic status [24]. Patients do not always see the relevance of quitting smoking, which suggests that our efforts to educate society about the negative influence of smoking is still not optimal. Another explanation might be that smokers feel stigmatised and isolated during CR as suggested by Beauchamp *et al. *[8]. Importantly, many authors warn that we should interpret the results of studies on smoking with caution, since in most studies ‘smoking’ was poorly defined [24]. Studies often relied on hospital records or self-reporting, without biochemical verification as we did in our current study. Since stop smoking rates are even lower when verified by objective biochemical verification we plea to continue this measurement in the future.

Since these were patients included in the OPTICARE trial, a potential bias from patients’ willingness to participate in a trial may exist. Certainly, the number of patients that drop-out may be higher in routine clinical care settings. Whether these patients have a different profile and/or cardiac outcome is not known. Also, our study population was at a relatively ‘low risk’ for drop-out, with relatively young patients without heart failure or renal impairment. Another limitation is that we do not know the reasons behind incomplete CR. From our own daily experience we know that this could be a wide variety of reasons, both medical and non-medical.

Finally, it is difficult to define what exactly constitutes ‘complete CR’ as it comprises a multidisciplinary programme, including exercise sessions, diet, smoking cessation and stress management courses. Each individual patient has different needs of attention, for instance attending dietary advise sessions may be more important for an obese diabetic patient than for a non-obese non-diabetic patient. All studies up to now focus on the number of exercise sessions: the effects of the other multidisciplinary sessions are still unknown and were therefore not incorporated in the current definition of complete CR. Future studies should address the importance of attending the non-exercise sessions.

## Conclusion

Post-ACS patients who did not complete a ‘standard’ 12-week CR programme had a higher incidence of adverse cardiac events during long-term follow-up than their counterparts who completed the programme. Persistent smokers are at risk of non-completion, which is, indirectly, an extra argument to motivate patients to quit smoking. Still, patients could not satisfactorily be identified as potential non-completers on the basis of their smoking behaviour alone. As CR is proven to be beneficial, further research is needed to understand the reasons why patients terminate prematurely.
